# Probiotic Potential of *Lactobacillus* Strains with Antifungal Activity Isolated from Animal Manure

**DOI:** 10.1155/2015/802570

**Published:** 2015-06-17

**Authors:** Soundharrajan Ilavenil, Hyung Soo Park, Mayakrishnan Vijayakumar, Mariadhas Valan Arasu, Da Hye Kim, Sivanesan Ravikumar, Ki Choon Choi

**Affiliations:** ^1^Grassland and Forage Division, National Institute of Animal Science, Rural Development Administration, Cheonan 330-801, Republic of Korea; ^2^Department of Botany and Microbiology, Addiriyah Chair for Environmental Studies, College of Science, King Saud University, Riyadh 11451, Saudi Arabia; ^3^The United Graduate School of Agricultural Sciences, Tottori University, Tottori 680-8553, Japan; ^4^Department of Biotechnology, PRIST University, Thanjavur, Tamil Nadu 613403, India

## Abstract

The aim of the study was to isolate and characterize the lactic acid bacteria (LAB) from animal manure. Among the thirty LAB strains, four strains, namely, KCC-25, KCC-26, KCC-27, and KCC-28, showed good cell growth and antifungal activity and were selected for further characterization. Biochemical and physiology properties of strains confirmed that the strains are related to the *Lactobacillus* sp.; further, the 16S rRNA sequencing confirmed 99.99% sequence similarity towards *Lactobacillus plantarum*. The strains exhibited susceptibility against commonly used antibiotics with negative hemolytic property. Strains KCC-25, KCC-26, KCC-27, and KCC-28 showed strong antifungal activity against *Aspergillus fumigatus, Penicillium chrysogenum, Penicillium roqueforti, Botrytis elliptica*, and *Fusarium oxysporum*, respectively. Fermentation studies noted that the strains were able to produce significant amount of lactic, acetic, and succinic acids. Further, the production of extracellular proteolytic and glycolytic enzymes, survival under low pH, bile salts, and gastric juice together with positive bile salt hydrolase (Bsh) activity, cholesterol lowering, cell surface hydrophobicity, and aggregation properties were the strains advantages. Thus, KCC-25, KCC-26, KCC-27, and KCC-28 could have the survival ability in the harsh condition of the digestive system in the gastrointestinal tract. In conclusion, novel *L. plantarum* KCC-25, KCC-26, KCC-27, and KCC-28 could be considered as potential antimicrobial probiotic strains.

## 1. Introduction

In the recent year, the consumers increased their demand for natural and chemical preservatives used in food industry to look for novel and alternative planning for the preservation of humans and animal foods. The term of probiotic is described as a feed supplement with live microbial culture. It can provide beneficial effects to host animals through improving its intestinal microbial balance and it can lead to great improvement in the intestinal microbial balance via enhancing nutrient absorption [1]. Further, it exhibits the potent antagonist effect on pathogens in GI tract [2]. Lactic acid bacteria produced essential antimicrobial metabolites that kill the other pathogenic bacteria [3]. There are numerous microorganisms that can be classified as probiotics belonging to the* Lactobacillus* and* Bifidobacterium* genera. Among them,* Lactobacilli* are considered an enteric organism in the industrial processing of fermented dairy, meat, vegetable, and cereal products. According to WHO (World Health Organization) for food to attain a probiotic status, microorganisms have to meet some of the principles related to their safety and biological properties. In terms of safety, the probiotic microorganism should not exhibit any pathogenic activity and should not be able to transfer antibiotic resistance genes and sustain genetic stability.

*Lactobacillus plantarum* is a lactic acid producing anaerobic bacteria, which are gram-positive, non-spore-producing fermentative bacteria. It is living in fermented foods and survives in gastrointestinal and urogenital tract in animals. Lactic acid bacteria do not exhibit any toxicity to the host animals. It is displaying the variety of positive physiological, biochemical, and genetic properties. These bacteria have the ability to produce the biologically important peptides, exopolysaccharides, secondary metabolites, and other organic compounds [4]. Lactic acid bacteria have a dominant role in the production of many fermented foods especially milk based products [5]. The genetically modified organisms have contributed to improving the numerous food products. However, it has a limitation for some reasons. So, the discovery of native strains from natural sources must be considered as the most suitable approach for identifying novel lactic acid bacteria. Therefore, we planned to isolate and characterize the wild lactic acid and evaluate their biological potentials* in vitro* model.

## 2. Materials and Methods

### 2.1. Sample Collection

The animal manure was collected from Cheonan, South Korea, in sterile propylene bags. Ten grams of the sample was mixed with 90 mL of sterile water and made a serial dilution up to 10^7^ and 100 *μ*L of sample was spread on the plate containing MRS (de Man, Rogosa, and Sharpe) media. Then, the plates were incubated at 30 ± 2°C for 24 to 48 h. Thirty colonies were picked up and their growth abilities in different juice such as barley, Corn, and Italian ryegrass and antifungal activity were analyzed. Based on growth and antifungal activity, we selected four strains named as KCC-25, KCC-26, KCC-27, and KCC-28 [6].

### 2.2. Biochemical and Physiological Characterization of Isolates

The biochemical test was analyzed according to methods described by Kozaki et al., 1992 [7]. The lactic acid, acetic acid, and succinic acid in MRS broth and juice such as Italian ryegrass, barley, Corn, and rye were estimated by UV-spectrophotometer (Tecan infinite m200) with Mega-Zyme assay kit. The API 50 CH test kit was used to analyze the carbohydrate test. The cellular enzymes were analyzed by api-ZYM kit (Biomeriux). The morphology of the isolate grown in the MRS medium was observed directly and by an inverted microscope (CKX41, Olympus Corporation, Tokyo, Japan).

### 2.3. Molecular Identification of Isolates

The DNA of the isolates was extracted using the commercial kit method (QlAamp DNA mini kit-Qiagen). The 16S rRNA amplified by PCR (Applied Biosystems-9700) using universal primers (27F AGA GTT TGA TCM TGG CTC AG and 1492R GTA TTA CCG CGG CTG CTG G) and was sequenced by Genetic Analyzer 3130 (Applied Biosystems, USA) in Solgent Co. Ltd. (16S rRNA, Seoul, Korea). The aligned 16S rRNA of the isolate was subjected to BLAST with the nonredundant database of NCBI Genbank. Based on the maximum identity score was selected and aligned using ClustalW multiple alignment software [8].

### 2.4. Antibiotic Sensitive Test for Isolates

Antibiotic sensitivity patterns were evaluated using the commercial kit method. Different types of the common antibiotic disc were loaded on the surface of the medium and kept at room temperature for 10 minutes. After that, plates were incubated at 30°C for 24 hours in the incubator. Then zone of inhibition was measured by a standard antibiotic disc chart [9].

### 2.5. Fungal Cultures and Preparations

Fungal strains such as* Aspergillus fumigatus* (KACC 40080),* Penicillium chrysogenum* (KACC 40399),* Penicillium roqueforti* (KACC 41354),* Botrytis elliptica* (KACC 43461), and* Fusarium oxysporum* (KACC 40051) were obtained from the Korean agricultural culture collection (KACC), South Korea. One-week-old conidial spore was prepared on potato dextrose agar plate with 0.05% Triton-X 100. The 10 *μ*L conidial suspension containing approximately 5 × 10^2^ conidia was used for the antifungal assay.

### 2.6. Screening of Antifungal Property of Isolates

#### 2.6.1. Pour Plat Method

24-hour grown isolates in MRS broth were loaded (discreet spot) on the solidified MRS agar plates. Then, the plates were incubated at 30 ± 2°C for 24 hours and then overlaid the potato dextrose medium (0.8%) containing 5 *μ*L of each conidial suspension on MRS media. Then, plates were incubated aerobically at 30 ± 2°C for a week and then the fungal growth was observed [6].

#### 2.6.2. Microdilution Method

Antifungal activities of isolates were analyzed by the method of Lavermicocca et al., 2003, with slight modification. Active isolates were inoculated in a 100 mL conical flask containing MRS broth and incubated at 30 ± 2°C for 48 hours with mild shaking. After 48 hours, the broth was centrifuged at 9000 rpm for 10 minutes at 4°C, and then the supernatant was collected and filtered through 0.22 *μ*M membrane filter. 190 *μ*L of isolates supernatant and 10% organic acids mixture in MRS broth (lactic acid, acetic acid, and succinic acid) were dispensed in 96 wells and then 10 *μ*L conidial suspensions were inoculated, without fermentative metabolites considered as a control. All the experimental plates were incubated at 30 ± 2°C for 72 hours. Fungal growth was measured at 600 nm using microplate reader [10]. Here, the growth of fungi in control was considered as a 100% growth. From that we have calculated the percentage of fungal growth inhibition.

### 2.7. Probiotic Property of Isolates

#### 2.7.1. pH Resistance

Different pH of the MRS broth was prepared with 1N HCl. Fresh cultures were inoculated onto different MRS broth and incubated at 30 ± 2°C for 3 hours. Aliquots cultures were withdrawn from each tube (0 h and 3 h after) and spread on the BCP and MRS media. The bacterial counts (CFU/mL) were recorded.

#### 2.7.2. Bile Salt Resistant

The fresh cultures were inoculated in MRS broth containing 0.3% of oxgall and 0.5% sodium deoxycholate (DCA) bile salts (Sigma, USA) and incubated at 30 ± 2°C. From that aliquots were withdrawn at different time intervals and the optical density was measured at 600 nm, without bile salt media considered as a control. The results were expressed as the growth of bacteria at 600 m in the presence of bile salts with respect to the control [9].

#### 2.7.3. Hemolytic, Bile Salt Hydrolase (Bsh) Activity, Hydrophobicity, and Aggregations Property of Isolates

Hemolytic activity was determined by streaking* L*.* plantarum* on the Colombia agar plates containing fresh blood and incubated for 24 h at 30 ± 2°C and then the hemolytic activity was examined. The* E*.* coli* was used as a control for hemolysis [11]. For Bsh activity, fresh* L*.* plantarum* was inoculated on the MRS media containing 0.5% sodium deoxycholate and it was incubated at 37°C for 72 h anaerobically. The presence of BSH activity showed white opaque colonies without bile salt considered as control [12].

For hydrophobicity, fresh* L*.* plantarum* grown in MRS broth was centrifuged at 1200 ×g for 10 min. After removal of the supernatant, the pellet was washed twice with PBS. Further, the pellet was resuspended in 5 mL of PBS. Taken 3 mL of* L. plantarum* suspension and 1 mL of xylene or chloroform were mixed by vortex and incubated at 37°C for 1 h for phase separations. The aqueous phase was taken gently to measure its absorbance at 600 nm and the % of hydrophobicity was calculated using the following calculation [13]:

(1) ΔOD=100×ODinitial−ODfinalODinitial=ΔODODinitial×100.

For aggregation property, freshly grown* L*.* plantarum* (4 × 10^8^ cells) in MRS was centrifuged at 12000 ×g for 10 min at 4°C and the pellet was collected and washed with PBS twice and then resuspended in 4 mL of PBS and the initial absorbance was read at 600 nm and then it was incubated for 3 h at 37°C. Aliquots (100 *μ*L) were withdrawn at a regular interval of 1 h from the top of the upper layer and mixed with 3.9 mL of PBS and the absorbance was read at 600 nm. The percentage of aggregation was calculated by following an equation: %AA = 1 − (*A*_*t*_/*A*_0_)*∗*100. *A*_*t*_ = absorbance at different time intervals (1, 2, and 3 h) and *A*_0_ is initial absorbance at 0 h. The coaggregation property was analyzed using* E*.* coli* as a pathogen [14].

#### 2.7.4. Gastric Juice Stress on Isolates

The gastric juice resistance property of isolated cultures was carried out against simulated gastric stress by the method of Charteris et al., 1998 [15]. The cholesterol assimilation property of* Lactobacillus* strains was determined [16].

#### 2.7.5. Statistical Analysis

All the numerical data were obtained from six independent experiments, and the analysis of these data was carried out with MS-Excel and SPSS 16 statistical analysis (SPSS Inc., Chicago, IL, USA). The results were presented as mean ± SD.

## 3. Results

### 3.1. Isolation and Morphology Characterization of Isolates

**Figure 1:**
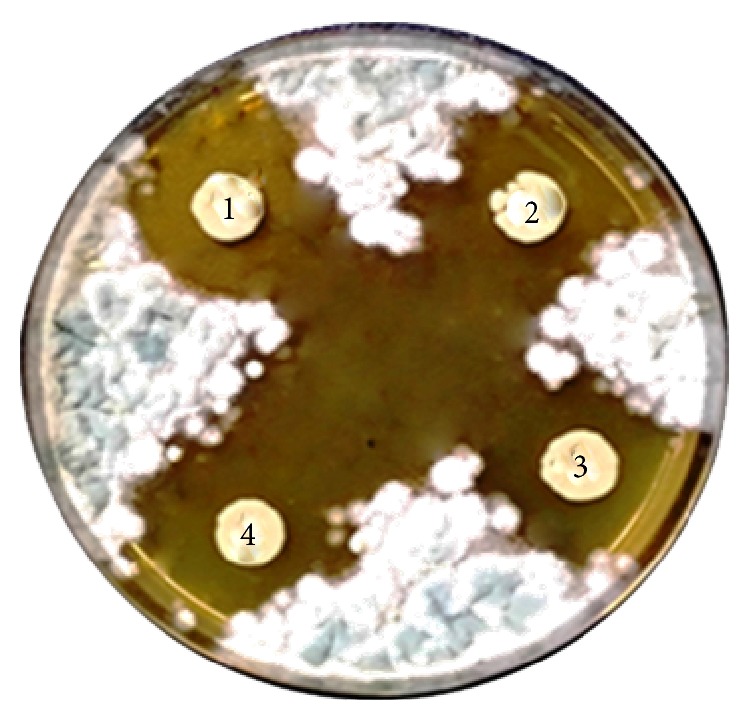
Antifungal activity of* L. plantarum* against* A. fumigatus.* 1: KCC-25, 2: KCC-26, 3: KCC-27, and 4: KCC-28.

Thirty* Lactobacillus* strains were isolated from animal manure that was collected from RDA farm, Cheonan, South Korea. All the* Lactobacillus* strains were screened for their antifungal activity against* A. fumigatus*,* P*.* chrysogenum*,* P. roqueforti*,* B. elliptica*, and* F. oxysporum*. Among thirty strains, 4 strains showed strong antifungal activity against* A. fumigatus*,* P*.* chrysogenum*,* P. roqueforti*,* B. elliptica*, and* F. oxysporum*, especially against* A. fumigatus* (Figure 1). These* Lactobacillus* strains are named as KCC-25, KCC-26, KCC-27, and KCC-28 (abbreviated from corresponding author name and colony number). The characteristics of* Lactobacillus* KCC-25, KCC-26, KCC-27, and KCC-28 were identified by biochemical and physiological characteristic as well as 16SrRNA gene sequences. The biochemical and physiological characteristics of strains exhibited gram-positive, creamy, nonmotile, rod-shaped catalase-negative, and mesophilic nature. It is suggested that these strains were related to the* Lactobacillus* sp. The* Lactobacillus* sp. KCC-25, KCC-26, KCC-27, and KCC-28 had ability to ferment the D-Arabinose, L-Arabinose, D-Ribose, D-Xylose, L-Xylose, D-Galactose, D-Glucose, D-Fructose, D-Mannose, D-Mannitol, Methyl-*α*-D-mannoside, and so forth and these strains are not able to ferment the glycerol, Methyl-*β*-D-xyloside, D-Adonitol, L-Sorbose and L-Rhamnose, and so forth (Table 1).

### 3.2. Antibiotic Sensitivity Test

The antibiotic sensitivity pattern analysis of* Lactobacillus* sp. KCC-25, KCC-26, KCC-27, and KCC-28 was tested against 15 different commonly used antibiotics. The strains exhibited susceptibility against commonly used antibiotics such as chloramphenicol, kanamycin, nitrofurantoin, tetracycline, streptomycin, colistin methanesulphonate, dicloxacillin, ampicillin, amikacin, cefoxitin, cefalexin, cefuroxime, and cotrimoxazole and it had resistance to sulphafurazole, gentamicin (Table 2).

### 3.3. Molecular Characterization of Isolates

The 16S rRNA gene of* Lactobacillus* sp. KCC-25, KCC-26, KCC-27, and KCC-28 was sequenced and their similarities using NCBI BLAST were analyzed. These data exhibited that these strains had 99.99% similarity to* Lactobacillus plantarum*. The 16S rRNA sequences of* L. plantarum* KCC-25,* L. plantarum* KCC-26,* L. plantarum* KCC-27, and* L. plantarum* KCC-28 were deposited into the NCBI Genbank database (accession numbers KP099604, KP099605, KP099606, and KP099607).

### 3.4. Fermentative Metabolite Productions

**Figure 2:**
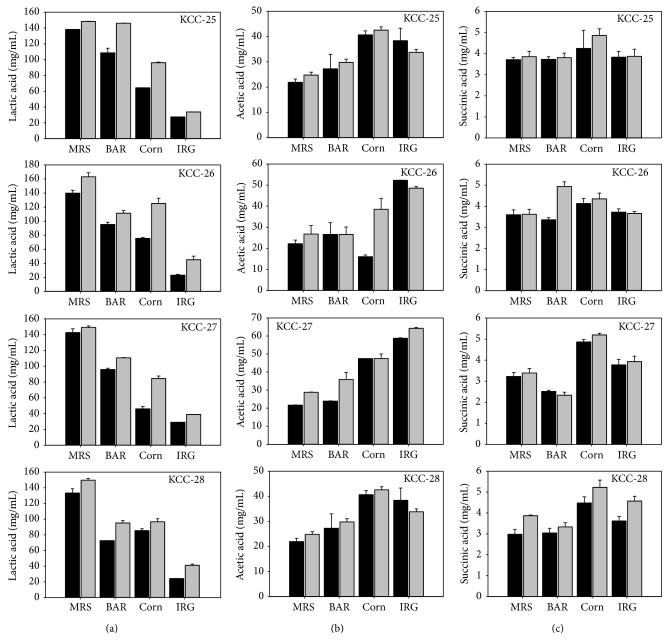
Fermentative metabolites of* L*.* plantarum* KCC-25, KCC-26, KCC-27, and KCC-28 in MRS broth, BAR (barley) juice, Corn juice, and IRG (Italian ryegrass) juice, under aerobic and microaerobic conditions. (a) Lactic acid (mg/mL), (b) acetic acid (mg/mL), and (c) succinic acid (mg/mL).

KCC-25, KCC-26, KCC-27, and KCC-28 produced significant amount of fermentative metabolites such as lactic acid, acetic acid, and succinic acid in MRS broth and different grass juice such as Italian ryegrass, Corn, barley, and rye. The maximum amounts of these acids were produced in KCC-26 as compared with other strains (Figure 2).

### 3.5. Antifungal Analysis of* L. plantarum*

Antifungal activity of KCC-25, KCC-26, KCC-27, and KCC-28 was analyzed against* A. fumigatus*,* P*.* chrysogenum*,* P. roqueforti*,* B. elliptica*, and* F. oxysporum*. All the spent media of* L. plantarum* exhibited strong antifungal activity against* A. fumigatus* as compared with* P*.* chrysogenum*,* P. roqueforti*,* B. elliptica*, and* F. oxysporum*. However, the remaining fungi were also inhibited by spent media of KCC-25, KCC-26, KCC-27, and KCC-28. Among the strains, KCC-26 exhibited best inhibitory activity against all tested fungi (Table 3). Then, we planned to find out a reason behind these inhibitions; therefore, we prepared MRS with 10% lactic acid, acetic acid, and succinic acid and then analyzed the antifungal activity against the same fungi. It showed significant antifungal activity against all tested fungi. It indicated that this inhibition may be due to fermentative metabolites produced by KCC-25, KCC-26, KCC-27, and KCC-28.

### 3.6. Probiotic Nature of* Lactobacillus plantarum*

**Figure 3:**
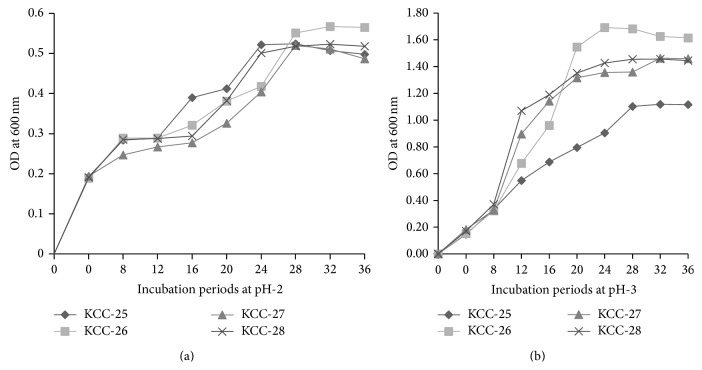
pH resistant properties of* L*.* plantarum* KCC-25, KCC-26, KCC-27, and KCC-28. (a)* L*.* plantarum* survival ability in pH-2. (b)* L. plantarum* survival ability in pH-3.

**Figure 4:**
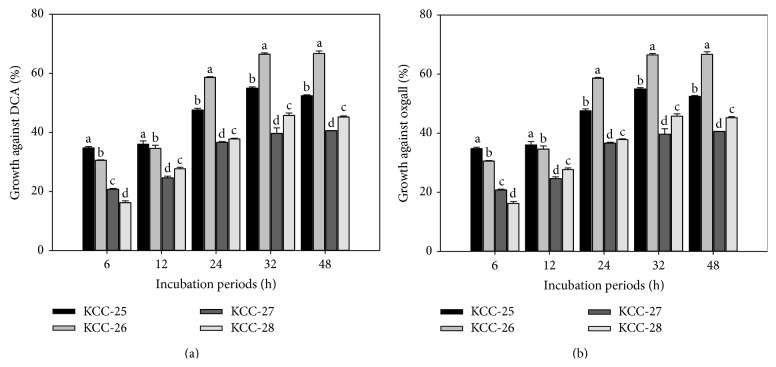
Bile salt tolerant properties of* L. plantarum*. (a) KCC-25, KCC-26, KCC-27, and KCC-28 growth in sodium deoxycholate (DCA). (b) KCC-25, KCC-26, KCC-27, and KCC-28 growth in oxgall. ^abcd^Different letters within treatment represent the significant difference (*p* < 0.05).

**Figure 5:**
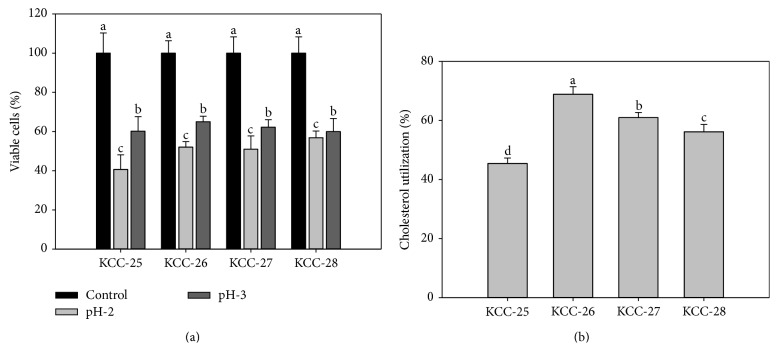
(a) Growth of KCC-25, KCC-26, KCC-27, and KCC-28 in gastric juice (pH-2 and pH-3) stress condition. (b) Cholesterol lowering property of* Lactobacillus* strains. ^abcd^Different letters within treatment represent significant difference (*p* < 0.05).

Low pH tolerance property of KCC-25, KCC-26, KCC-27, and KCC-28 was investigated by culturing KCC-25, KCC-26, KCC-27, and KCC-28 at different pH (2 and 3). The marginal reduction of the bacterial growth was observed at pH-2 and pH-3. It indicated that these strains had the ability to grow in low pH (Figures 3(a)-3(b)). KCC-25, KCC-26, KCC-27, and KCC-28 were supplemented with 0.3% oxgall and 0.5% sodium deoxycholate for 3 h showed a marginal decrease of % of bacterial viability as compared with control (Figures 4(a)-4(b)). In addition, the KCC-25, KCC-26, KCC-27, and KCC-28 exposed to the artificial gastric juice of pH-2 and pH-3 with pepsin for 3 h reduced the % of bacterial viability as compared with control bacteria. However, a significant % of KCC-25, KCC-26, KCC-27, and KCC-28 were surviving in the harsh gastric juice conditions (Figure 5(a)). All these strains had significant ability to survive in the low pH, bile salts, and gastric juice. These strains had the ability to utilize the cholesterol from MRS broth after 24 h incubation (Figure 5(b)). The maximum level of cholesterol was assimilated by KCC-26 as compared with other strains. The* Lactobacillus* sp. KCC-25, KCC-26, KCC-27, and KCC-28 produced different kind of extracellular enzymes such as *β*-Galactosidase, *α*-Galactosidase, *α*-Glucosidase, *β*-Glucosidase, leucine arylamidase, valine arylamidase, acid phosphatase, naphthol-AS-biphosphohydrolase, trypsin-like serine protease, and other enzymes (Table 4). All the strains showed significant hydrophobicity and aggregations property (Table 5).

## 4. Discussion

The lactic acid bacteria (LAB) are very important for developing food products, promoting the quality of products [17]. LAB were used as starter cultures in the development of fermented products. Probiotic applications of* Lactobacillus* strains were characterized by Pascual et al., 2008 [18]. The application of genetically modified organism to improve the properties of starter cultures, however, for fermented foods remains to be limited for a number of reasons. Therefore, the discovery of new wild strains is considered as the most suitable approach for making novel lactic acid bacteria [19]. In the present study, we isolated 30 strains from animal manure. Based on the cell growth and antifungal efficiency, we selected four lactic acid bacteria for further investigation. These strains are initially named as KCC-25, KCC-26, KCC-27, and KCC-28. They displayed significant antifungal activity against* A. fumigatus*,* P*.* chrysogenum*,* P. roqueforti*,* B. elliptica*, and* F. oxysporum*; particularly, KCC-26 showed strong antifungal activity against all the tested fungi. The maximum % of fungal inhibition activity was observed against* A. fumigatus* by all the strains. Similarly, Arasu et al., 2013 [6], reported that the* Lactobacillus plantarum* K-46 showed potent antifungal activity against fungi* G. moniliformis* and* A. fumigatus* and often it caused spoilage in bakery products [20]. The lactic acid bacteria KCC-3 and KCC-24 inhibit growth of* A. fumigatus*,* P*.* chrysogenum*,* P. roqueforti*,* B. elliptica*, and* F. oxysporum* by their secondary metabolites [9, 21]. Antifungal activities were depending on microbial community composition as well as environment and growth conditions [22]. The microbes producing amazing primary and secondary metabolites are acting as best antibiotics, antitumor agents and pesticides, and so forth [23]. Antimicrobial metabolites produced from both microbes are usually acting as competitive weapons between the microbes [24]. In the present study, all the strains produced a significant amount of lactic acid, acetic acid, and succinic acid. The maximum amount of fermentative metabolites was produced in* L. plantarum* KCC-26. Antifungal activity of these strains may be due to its fermentative metabolites. Okkers et al., 1999 [25], reported the supernatant of LAB having protein-like compounds which inhibit fungal growth. However, in our study, we confirmed the main reasons for these inhibitions are due to fermentative metabolites containing organic acids such as lactic acid, acetic acid, and succinic acid. In addition, all the* L. plantarum* were highly sensitive towards almost all tested antibiotics, which suggested that there is no pathogenicity. Therefore, these strains could be considered as safe and potent probiotic. The probiotic bacteria must provide safety because they do not give any harmful effects to the host. In the present study, the KCC-25, KCC-26, KCC-27, and KCC-28 did not show hemolytic activity in the human blood. However, the positive strain* E*.* coli* showed hemolytic activity. Therefore, all strains are considered as safe organism and could be used as probiotic strains. This result was concurrent with previous reports of Gao et al., 2012 [26].

*β*-Glucosidase is an important enzyme that plays a main role in the conversion of glycosides into aglycones and it is easily absorbed by the intestine; therefore, it is considered as biologically more active form than the glycosides [27]. The *β*-Galactosidase acts upon *α* 1, 4 bonds breakdown of starch molecules and disaccharide to Glucose [28]. Milk casein hydrolyzed into small peptides and free amino acids via an action of bacterial protease and peptidases, which plays the main role in the chess ripening industries [29]. Our strains had the ability to produce the different kind of extracellular enzymes. All isolates produced *β*-Galactosidase that is essential enzyme for hydrolysis of lactose into Galactose and Glucose. The probiotic bacteria utilize lactose and convert it into SCFA (short chain fatty acids). It is beneficial for the host [30]. Any products fermented with *β*-Galactosidase producing microbes play an important role for lactose intolerance treatment [31].

Survival and behavioral properties of strains under the harsh condition of GIT (gastrointestinal tract) are important criteria for selection of probiotic strains, because these strains must survive under the conditions from mouth to GIT. Saliva contains lysozyme, which kills the bacteria; probiotic microbes must need to survive in low pH tolerance because in stomach pH range is between 1.5 and 3.0 and finally, intestinal part contains different kind of bile salts which are toxic to the microbes [32]. So, potent probiotic strains must survive in these conditions. In the present study, these strains had the ability to survive in all the conditions. It is confirmed by low pH tolerance, bile salt resistance, and gastric juice stress analysis on* L. plantarum* KCC-25, KCC-26, KCC-27, and KCC-28. Among these, KCC-26 showed increased % of growth against bile salts such as oxgall and sodium deoxycholate. It indicated that these strains could survive in all the conditions in the digestive systems. KCC-25, KCC-26, KCC-27, and KCC-28 lowered cholesterol level in the MRS broth. KCC-26 showed maximum cholesterol assimilation as compared with other strains. Bsh positive organisms are considered as potent probiotic organisms as compared to negative Bsh organisms because Bsh have better ability to remove cholesterol [33]. In the present study, we found all the strains had the ability to remove cholesterol due to its Bsh activity. The conjugated bile salts are important for cholesterol absorption from intestine. The Bsh enzyme produced by probiotic bacteria hydrolyzed conjugated bile salt, which prevents the cholesterol absorption from intestine and enhances the cholesterol assimilation by the probiotic bacteria [34].

Microbial adhesions to hydrocarbons are called MATH test. It is used to find out the microbial cell surface hydrophobicity. Bacterial cells exhibiting more hydrophobicity are expected to make potent interaction with mucus or epithelial cells [35, 36]. In our study, all strains showed significant hydrophobicity with xylene and chloroform. Particularly, KCC-26 exhibited higher hydrophobicity than the other strains. It suggests that these strains had strong hydrophobic interaction and make an adhesion to the mucus or epithelial cells through hydrophobic interaction.

The ability of microorganism to adhere and colonise in epithelial cells and mucosal surface is an important key for selection of probiotic [37]. The autoaggregation has an essential role in biofilm formation which will help with intestinal colonization and bind effectively to intestinal epithelium which prevents pathogen adhesions [38]. In the present study, all strains exhibited significant aggregation; particularly KCC-26 exhibited greater autoaggregation than the other strains, and it reveals clumping of cells. In coaggregation, one microorganism adheres with other organisms. The coaggregation with a pathogen allows the probiotic bacteria to produce antimicrobial agents that may inhibit the growth of pathogenic microorganisms in the gastrointestinal tract [39]. Among these strains, KCC-26 exhibited higher aggregates with* E*.* coli.* The aggregation property for the probiotic bacteria may be due to extracellular polysaccharide found in the cell surface [40].

## 5. Conclusion

We isolated and identified four antifungal* Lactobacillus plantarum* KCC-25–KCC-28 from animal manure samples and they were assessed for their probiotic potentials. These strains showed potent antifungal activity against* A. fumigatus*,* P*.* chrysogenum, P. roqueforti*,* B. elliptica*, and* F. oxysporum*. These strains produced the significant amount of lactic acid, acetic acid, and succinic acid that could be responsible for the antifungal activity. In addition, all these strains have susceptibility against most of the antibiotics and did not exhibit the hemolytic activity on human blood, so these strains are nontoxic to host and can be considered safe. Further, it survived against low pH, bile salts, and artificial gastric stress. Also, these strains showed Bsh positive, cholesterol lowering nature, cell surface hydrophobicity, and aggregation property. Overall results suggested that these strains could be used as potent probiotic strains.

## Figures and Tables

**Table 1:** Biochemical characterizations of isolated strains by API 50 CHL system.

Name of carbohydrates	KCC-25	KCC-26	KCC-27	KCC-28
Glycerol	−	−	−	+
Erythritol	−	−	−	+
D-Arabinose	+	+	+	+
L-Arabinose	+	+	+	+
D-Ribose	+	+	+	+
D-Xylose	+	+	+	+
L-Xylose	+	+	+	+
D-Adonitol	−	−	−	−
Methyl-*β*-D-xyloside	−	−	−	−
D-Galactose	+	+	+	+
D-Glucose	+	+	+	+
D-Fructose	+	+	+	+
D-Mannose	+	+	+	+
L-Sorbose	−	−	−	−
L-Rhamnose	−	−	−	−
Dulcitol	−	−	−	−
Inositol	−	−	−	−
D-Mannitol	+	+	+	+
D-Sorbitol	−	−	−	−
Methyl-*α*-D-mannoside	+	+	+	+
Methyl-*α*-D-glucoside	−	−	−	−
N-Acetyl glucosamine	+	+	+	+
Amygdalin	+	+	+	+
Arbutin	+	+	+	+
Esculin ferric citrate	+	+	+	+
Salicin	+	+	+	+
D-Cellobiose	+	+	+	+
D-Maltose	+	+	+	+
D-Lactose	+	+	+	+
D-Melibiose	+	+	+	+
D-Saccharose	+	+	+	+
D-Trehalose	+	+	+	+
Inulin	−	−	−	−
D-Melezitose	+	+	+	+
D-Raffinose	+	+	+	−
Amidon	−	−	−	−
Glycogen	−	−	+	−
Xylitol	−	−	−	+
Gentiobiose	−	−	−	−
D-Turanose	+	+	+	−
D-Lyxose	+	+	+	−
D-Tagatose	−	−	+	−
D-Fucose	−	−	+	−
L-Fucose	−	−	+	−
D-Arabitol	−	−	−	−
L-Arabitol	−	−	−	−
Potassium gluconate	−	−	−	−
Potassium 2-ketogluconate	+	+	+	−
Potassium 5-ketogluconate	+	+	+	+

+: positive response; −: negative response.

**Table 2:** Antibiotic sensitivity of *Lactobacillus plantarum*.

S. number	Name of antibiotics	Concentrations (*μ*g)	KCC-25	KCC-26	KCC-27	KCC-28
1	Chloramphenicol (C)	50	S	S	S	S
2	Kanamycin (K)	30	S	S	S	S
3	Nitrofurantoin (NIT)	50	S	S	S	S
4	Tetracycline (TE)	100	S	S	S	S
5	Streptomycin (S)	25	S	S	S	S
6	Sulphafurazole (SF)	300	R	R	R	R
7	Colistin methanesulphonate	100	S	S	S	S
8	Dicloxacillin (D/C)	1	S	S	S	S
9	Ampicillin (AMP)	10	S	S	S	S
10	Amikacin (AK)	30	S	S	S	S
11	Gentamicin (GEN)	10	R	R	R	R
12	Cefoxitin (CX)	30	S	S	S	S
13	Cefalexin (CN)	30	S	S	S	S
14	Cefuroxime (CXM)	30	S	S	S	S
15	Cotrimoxazole (COT)	25	S	S	S	S

S: susceptibility; R: resistant.

**Table 3:** Percentage of fungal inhibition by fermentative metabolites of KCC-25, KCC-26, KCC-27, and KCC-28.

Tested fungi	KCC-25^1^	KCC-26^1^	KCC-27^1^	KCC-28^1^	C1 medium^2^
*A. fumigatus *	63.22 ± 0.8	76.02 ± 2.0	68.27 ± 1.1	60.82 ± 1.9	57.32 ± 2.51
*P. chrysogenum *	56.94 ± 1.3	69.07 ± 0.4	65.65 ± 0.8	52.10 ± 0.2	52.08 ± 1.52
*P. roqueforti *	60.51 ± 0.1	73.14 ± 5.3	53.67 ± 1.2	53.34 ± 1.9	48.46 ± 2.25
*B. elliptica *	25.80 ± 1.1	49.68 ± 1.4	25.24 ± 0.9	26.26 ± 1.8	46.32 ± 2.65
*F. oxysporum *	56.01 ± 1.3	68.01 ± 1.5	52.59 ± 0.3	52.24 ± 0.3	51.42 ± 2.96

^1^Fermentative metabolites of *Lactobacillus plantarum*. ^2^C1 medium containing 10% organic acids. The percentage of fungal growth inhibition was calculated from fungal growth in control. The results were expressed as mean ± SD of three replicates.

**Table 4:** Extracellular enzymes production by isolated strains.

Extracellular enzymes	KCC-25	KCC-26	KCC-27	KCC-28
Alkaline phosphatase	+++	++	++	+++
Esterase (C_4_)	++	++	+	+++
Esterase lipase (C_8_)	++	++	+	++
Lipase (C_14_)	++	++	+	++
Leucine arylamidase	+++	+++	+++	+++
Valine arylamidase	+++	+++	+++	+++
Cystine arylamidase	++	++	++	+++
Trypsin-like serine protease	++	++	++	++
*α*-Chymotrypsin	++	++	+	+
Acid phosphatase	+++	+++	+++	+++
Naphthol-AS-biphosphohydrolase	+++	+++	++	+++
*α*-Galactosidase	++	+++	++	++
*β*-Galactosidase	+++	+++	+++	+++
*β*-Glucuronidase	++	++	++	++
*α*-Glucosidase	+++	+++	+++	+++
*β*-Glucosidase	+++	+++	++	+++
N-Acetyl-*β*-glucosaminidase	+++	+++	+++	+++
*α*-Mannosidase	+	+++	++	+
*α*-Fucosidase	+	+	+	+

+++: strong production; ++: moderate production; +: less production.

**Table 5:** Cell surface hydrophobicity and aggregation properties of *Lactobacillus* strains.

Parameters (%)	KCC-25		KCC-26		KCC-27		KCC-28	
	Xylene	Chloroform	Xylene	Chloroform	Xylene	Chloroform	Xylene	Chloroform
Hydrophobicity	27.52 ± 1.2	15.26 ± 0.8	38.27 ± 0.7	21.578 ± 1.2	32.15 ± 0.9	18.24 ± 0.25	34.82 ± 0.1	21.54 ± 0.7

Autoaggregation	1 h	20.14 ± 0.14	26.41 ± 0.52		21.24 ± 1.02		23.57 ± 1.02	
	2 h	56.12 ± 1.25	68.21 ± 0.51		63.15 ± 1.08		64.20 ± 0.52	
	3 h	58.61 ± 1.30	72.14 ± 0.58		65.27 ± 1.52		68.26 ± 0.96	

Coaggregation	l h	03.80 ± 0.08	06.2 ± 0.560		02.70 ± 0.07		02.30 ± 0.05	
	2 h	08.24 ± 0.07	12.65 ± 0.58		07.85 ± 0.05		09.23 ± 0.01	
	3 h	10.23 ± 0.05	17.68 ± 0.62		10.24 ± 0.01		12.05 ± 0.07	
